# Wildfire-Derived Pyrogenic Carbon Modulates Riverine Organic Matter and Biofilm Enzyme Activities in an In Situ Flume Experiment

**DOI:** 10.1021/acsestwater.1c00185

**Published:** 2021-06-25

**Authors:** Lukas Thuile Bistarelli, Caroline Poyntner, Cristina Santín, Stefan Helmut Doerr, Matthew V. Talluto, Gabriel Singer, Gabriel Sigmund

**Affiliations:** † Institute of Ecology, University of Innsbruck, Technikerstraße 25, 6020 Innsbruck, Austria; ‡ Institute of Microbiology, University of Innsbruck, Technikerstraße 25, 6020 Innsbruck, Austria; § Research Unit of Biodiversity, Spanish National Research Council (CSIC), E-33600 Mieres, Spain; ∥ Department of Biosciences, Swansea University, Singleton Park, Swansea SA2 8PP, U.K.; ⊥ Department of Geography, Swansea University, Singleton Park, Swansea SA2 8PP, U.K.; # Department of Environmental Geosciences, Centre for Microbiology and Environmental Systems Science, University of Vienna, Althanstraße 14, 1090 Wien, Austria

**Keywords:** charcoal, black carbon, dissolved organic carbon, dissolved organic matter, biofilm functioning

## Abstract

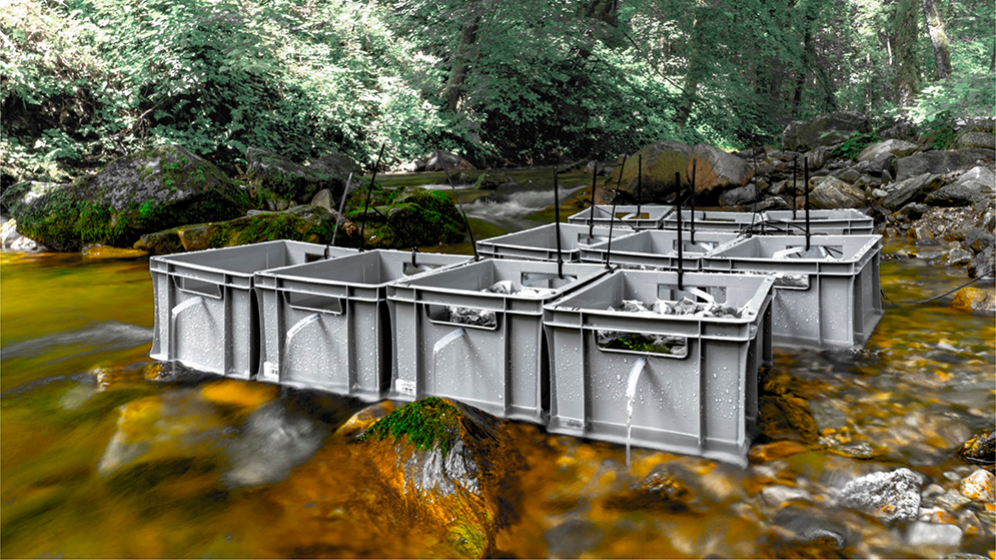

Wildfires produce large amounts of pyrogenic carbon (PyC), including charcoal, known for its chemical recalcitrance and sorption affinity for organic molecules. Wildfire-derived PyC can be transported to fluvial networks. Here it may alter the dissolved organic matter (DOM) concentration and composition as well as microbial biofilm functioning. Effects of PyC on carbon cycling in freshwater ecosystems remain poorly investigated. Employing in-stream flumes with a control versus treatment design (PyC pulse addition), we present evidence that field-aged PyC inputs to rivers can increase the dissolved organic carbon (DOC) concentration and alter the DOM composition. DOM fluorescence components were not affected by PyC. The in-stream DOM composition was altered due to leaching of pyrogenic DOM from PyC and possibly concurrent sorption of riverine DOM to PyC. Decreased DOM aromaticity indicated by a lower SUVA_245_ (−0.31 unit) and a higher pH (0.25 unit) was associated with changes in enzymatic activities in benthic biofilms, including a lower recalcitrance index (β-glucosidase/phenol oxidase), suggesting preferential usage of recalcitrant over readily available DOM by biofilms. The deposition of particulate PyC onto biofilms may further modulate the impacts of PyC due to direct contact with the biofilm matrix. This study highlights the importance of PyC for in-stream biogeochemical organic matter cycling in fire-affected watersheds.

## Abstract 2

Pyrogenic carbon alters organic matter and pH in streams, affecting microbial functioning with potential implications for in-stream carbon cycling.

## 1 Introduction

Vegetation fires annually burn ∼4% of Earth’s vegetated land surface, forming approximately 256 Tg of pyrogenic carbon (PyC).^1^ PyC is a continuum of thermally altered organic materials, of which a considerable fraction is highly recalcitrant, persisting in the environment for prolonged periods of time.^2^ The chemical composition of PyC is determined by fire conditions (e.g., temperature, charring duration, and oxygen availability) and fuel type (e.g., grassy vs woody).^3^ In fire-affected landscapes, fresh as well as aged PyC, in both dissolved and particulate forms, is mobilized to fluvial ecosystems via water erosion.^4−6^ Indeed, the significant presence of charcoal in river bed deposits in fire-affected ecosystems was the subject of investigation decades ago^7^ and hydrological events (e.g., stormwater runoff) can transport large quantities of PyC into river ecosystems in short periods of time.^6,8^ Coppola and colleagues^9^ found that globally 15.8 ± 0.9% of riverine particulate organic carbon is of pyrogenic origin. Jones and colleagues^10^ estimated that PyC accounts for 12 ± 5% of the riverine dissolved organic carbon (DOC, i.e., filtered <0.45 μm). PyC is, therefore, a quantitatively substantial fraction of the organic matter component in many river systems.

Rivers not only transport but also transform organic matter on its way downstream toward the ocean. These transformations can occur via photochemical, microbial, and mechanical processes.^11−14^ Photochemical reactions, which are strongly dependent on the site,^15^ preferentially degrade highly aromatic dissolved organic matter (DOM) derived from PyC.^16^ In-stream microbiota, especially biofilm communities that are hot spots of microbial functioning, are central to the role of fluvial ecosystems as bioreactors of terrestrial material,^17^ but the microbial degradation of pyrogenic DOM remains poorly understood. Bostick and colleagues^18^ recently measured considerable degradation of the labile DOC fraction leached from fresh PyC in laboratory-based experiments; however, the metabolization of fresh as well as aged PyC under natural conditions remains elusive. Finally, mechanical processes can physically disintegrate PyC into smaller particles.^14^ This can lead to leaching of particulate as well as dissolved pyrogenic organic matter and metals.^14,19,20^

The less aromatic, more labile fraction of PyC can be a relevant component of in-stream carbon turnover.^21,22^ Changes in DOC quantity and DOM composition induced by PyC may alter microbial functioning, on the basis of observations in non-fire-affected aquatic systems.^12,23,24^ For example, Freixa and colleagues^24^ showed that shifts in DOM sources (i.e., allochthonous to autochthonous) along the river continuum were accompanied by a change in extracellular enzymatic activities. In addition to pyrogenic DOM, PyC particles can also affect DOM composition and its bioaccessibility by interacting with riverine DOM via selective adsorption. This process has previously been observed for other carbonaceous materials, including carbon nanomaterials,^25,26^ graphite, and biochar.^26^

PyC is therefore a common component in limnic systems, with the potential to alter riverine microbial DOM cycling. However, field-based experiments for elucidating specific impacts and processes are still lacking. Here we carry out a field experiment in a natural river to investigate the effects of wildfire-derived PyC inputs on in-stream DOM properties and biofilm functioning. In addition, to the best of our knowledge this study is the first to analyze effects of PyC on in-stream biofilm enzymatic activities. We hypothesized that PyC would affect (i) in-stream DOM composition and DOC concentration due to sorption of riverine DOM and leaching of pyrogenic DOM leading to a net increase in DOC concentration and (ii) microbial functions, measured via enzymatic activities by altering substrate composition and pH.

## 2 Methods

### 2.1 Site Description and Field Experimental Design

This study was carried out at the Austrian river Kleine Ysper (latitude 48.218N, longitude 15.023E), a tributary of the Ysper with a slope of 3.3 cm/m, an average width of 5.47 ± 1.44 m, and an upstream catchment area of 68.30 km^2^ at a site situated ∼4 km upstream of the confluence with the Danube. This site was selected because the land use in its catchment area is dominated by mixed forest and seminatural areas that are widespread in the region, and the site was characterized in a previous study.^27^ To the best of our knowledge, no wildfire occurrences have been recorded in the upstream catchment area, making this a pristine site for the experiment. Atmospheric PyC inputs are possible, but we found no evidence thereof. The used PyC was selected as a proxy for forest fire-derived PyC. The PyC was charcoal consisting of fully charred woody material from *Pinus sylvestris*, collected from the ground as pieces with a radius of 0.5–2.5 cm one year after an extensive wildfire in a pine forest (Karbole, Sweden). This charcoal was produced at an estimated maximum temperature of 800 °C and a charring duration of ≥200 s.^28^ Particles were gently crushed, sieved to 0.5–1.0 cm, and homogenized. The PyC’s molar carbon/nitrogen (C/N) ratio was measured in triplicate on a CHNS analyzer (Vario MACRO, Elementar).

**Figure 1:**
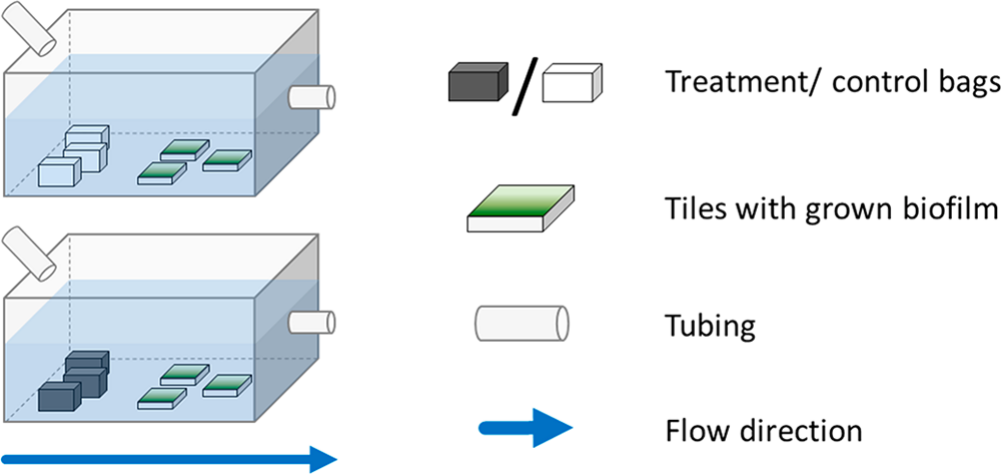
Stream-side flume setup. Flume design showing bags filled with inert quartz sand (top) and PyC (bottom) for control and treatment, respectively.

For the experiment, 60 ceramic tiles (25 cm^2^) fixed to a wooden board were exposed on the riverbed from June 14, 2019, to July 12, 2019, for colonization by native biofilm communities. On July 12, 2019, a total of 10 experimental in situ flumes built from commercially available 20 L boxes were placed in the river. Inflow of river water into the flumes was provided using 6 m long tubing (8 mm inner Ø) for each box. The inflow of each tube was placed 5 m upstream of the flumes at a hydraulic head of 20 cm, resulting in an average discharge of 0.47 ± 0.01 L min^–1^, which corresponds to a total volume of approximately 225 L passing through each flume over the duration of the experiment (8 h). The water volume in each box was approximately 15 L. A WTW probe was used during each sampling to monitor the pH, conductivity, and oxygen concentration in each flume. Biofilm-carrying tiles from the river were randomly distributed across the flumes (Figure 1). Five flumes were used as treatment and control flumes. Fifteen paper-filter bags, each containing 15 g of particulate PyC, were added to each treatment flume at the start of the experiment. The amount of PyC added was arbitrarily chosen as a potential postfire scenario. Actual amounts would depend on site factors such as fire behavior, amount and type of fuel affected, slope steepness, and size of the hydrological event.^6,8^ The filter bags had a mesh size <50 μm to allow the release of small PyC particles and DOC. In the control flumes, filter bags without PyC were added to account for potential interactions of riverine DOM with the filter bag.

Water samples were collected at each outflow 1, 2, 3, 4, 6, and 8 h after the beginning of the experiment. Additionally, we monitored O_2_, pH, and conductivity using a WTW probe (Xylem). At 1, 4, and 8 h, samples were taken for the measurement of total concentrations of Li, Na, Mg, Al, Ca, Sc, Mn, Fe, Co, Zn, Rb, Sr, Y, Mo, Ba, La, Ce, Pr, Nd, Sm, Gd, Dy, Pb, and U (see Figure S1 and Table S1 for details).

To assess the deposition of particulate PyC on biofilm surfaces via light microscopic analysis, 20 microscopic glass slides were placed at the bottom of the flumes for the duration of the experiment (see the Supporting Information for details).

### 2.2 PyC Leaching Experiment

Due to deposition and partial dilution, it was not possible to quantify the leached particulate organic carbon fraction in the flume setup. To overcome this issue, we performed complementary leaching experiments in the laboratory for the duration of the flume experiment using river water as well as Milli-Q water. The proportion of PyC and filter bag to water (15 g of PyC/L) used in the flume experiments was reproduced in 1 L Schott bottles that were gently agitated with a magnetic stirrer for the same duration that was used in the flume experiment (8 h, three replicates). Filter bags were removed from the bottles, and the suspension was shaken to obtain well-intermixed water samples for DOC, DOM properties, and total organic carbon (TOC) measurements. Total carbon (TC) and inorganic carbon (IC) were measured to calculate TOC (TOC = TC – IC) on a TOC-L analyzer (Shimadzu) using 40 mL vials with a magnetic stirrer to avoid deposition.

### 2.3 Properties of Organic Matter

To determine the DOC concentration and DOM properties, water samples were sterile filtered through prewashed 0.2 μm Minisart syringe filters (Sartorius). For DOC measurements, we filtered 2 mL of sample into an HPLC vial that was acidified to pH 2 with ultrapure HCl. DOC was analyzed by high-temperature combustion on a multi N/C 2100s instrument (Analytik Jena) with a limit of quantification of 50 μg L^–1^ and a coefficient of variation of 1–2%. DOM samples were filtered without any treatment into 10 mL glass vials with PTFE septa. The fluorescent and light absorbing moieties of DOM samples were analyzed spectrofluorometrically on a Horiba Aqualog (Horiba Ltd.), which measures absorbance (250–600 nm, 5 nm increment) and excitation–emission matrices (EEMs, excitation at 250–550 nm, emission at 250–600 nm, 5 nm increment) concomitantly using a 1 cm quartz cuvette and Milli-Q water as an optical blank.

We applied absorbance-based measurements to additionally cover DOM components that absorb light but are not fluorescent. The decadal absorption coefficient at 254 nm was used to compute specific UV absorption (SUVA_254_), which commonly serves as a proxy for aromaticity.^29^ Rayleigh scatter was deleted from EEMs, and Raman scatter was removed by subtracting MQ EEMs from sample EEMs.^30,31^ All EEMs were used for parallel factor analysis (PARAFAC)^32,33^ using the R package staRdom.^34^ After exclusion of outliers using the leverage() function in the staRdom routine, 54 EEMs were used to derive four fluorescent components, which were compared with data from the literature using the online database OpenFluor.

### 2.4 Potential Extracellular Enzymatic Activity

Biofilm grown on the submerged tiles was used to perform the potential extracellular enzymatic activity (EEAs) assays reflecting the maximum capacity of an enzyme to cleave a given substrate. We measured the activity of the enzymes β-glucosidase (EC 3.2.1.21), β-xylosidase (EC 3.2.1.37), cellobiohydrolase (EC 3.2.1.91), β-*N*-acetylglucosaminidase (EC 3.2.1.30), phosphatase (EC 3.1.3.1–2), lipase (EC 3.1.1.3), leucine-aminopeptidase (EC 3.4.11.1), and phenol oxidase (EC 1.10.3). These enzymes are broadly used to understand the effects of DOM on bacterial community functioning.^24,35−42^ Enzyme assays were prepared beforehand in deep well plates and brought to the field frozen. At the end of the 8 h field experiment, we scraped off the biofilm from the submerged tiles using a scalpel and subsequently homogenized it using a frother. An equal amount (300 μL) of biofilm slurry was used to inoculate each well of the assay, which was incubated for 1 h in the dark at the river temperature. Thereafter, the process was stopped using buffers, and plates were immediately frozen on site. After 2 days, the plates were thawed, gently centrifuged, and analyzed in the laboratory on a Spark plate reader (Tecan Trading AG). Measured enzymatic activities were used to compute the following enzymatic activity ratios (ERs) as these are independent of biomass: Xyl/Glu, which indicates increased use of large polymeric carbon compounds (e.g., derived from plant material); (Glu + Xyl)/Cbh, which indicates the use of readily available polysaccharides over complex polysaccharides; Glu/Pep, which indicates a prevalent use of glucose rather than amino acids as the primary carbon source; Glu/Pox, also called the recalcitrant index, which indicates the use of readily available material over more complex lignin-derived material; Pep/Pho, which indicates whether an ecosystem is N-limited rather than P-limited; and NAG/Pox, which indicates the use of readily available C and N over the use of lignin-derived C.^36,37^ Further experimental details can be found in the Supporting Information.

### 2.5 Statistical Analysis

To analyze DOC and DOM data, we applied Gaussian process (GP) regressions (the code and data can be found at https://github.com/lukastb/LimnicFires) as they are able to account for the non-independence in the response variables that results from the repeated measurements over time. GP regression employs a flexible model structure that can describe the effects of predictors on both the mean and the (auto-) covariance structure of the response variable.^43^ We employed a simple linear equation for the mean function:
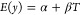
1where *T* is an indicator variable indicating whether the treatment was applied; with no covariance and constant variance, this model is nearly identical to a simple linear regression with a categorical predictor.

We modeled covariance within flumes as a decaying function of observation time using a Gaussian kernel function:
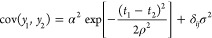
2where α and ρ are hyperparameters controlling the variance and bandwidth of the Gaussian process, respectively, *δ*_*ij*_ is an indicator that is 1 if *i* = *j* and 0 otherwise, σ^2^ is the residual variance of response variable *y*, and *t* is the time of measurement. This kernel generates a variance–covariance matrix in which covariance increases as the time between a pair of observations decreases, thus accounting for any autocorrelation due to the repeated measures design of the experiment. We assumed covariance among flumes was zero, and we calibrated a single set of hyperparameters (i.e., α, ρ, and σ) for all flumes, thus assuming that the strength of the time–covariance relationship was identical among flumes. We calibrated the model using the package rstan.^44^ We also compared the results of the GPs to generalized linear models (GLMs) with no time dependence and obtained similar parameter estimates. Differences between enzyme ratios in treatment and control flumes were analyzed using *t* tests as these data were collected exclusively at the end of the experiment. All uncertainty estimates are provided as standard errors.

## 3 Results and Discussion

### 3.1 Pyrogenic Carbon Increases pH and Dissolved Organic Carbon

The pH was increased by approximately 0.25 unit in the treatment flumes compared to the controls after the first 3 h of the experiment and remained nearly constant throughout the remaining 5 h (see Figure S2a). In contrast, O_2_ content and conductivity were not strongly affected by PyC addition (see Figure S2). This is in good agreement with the well-documented alkalinity of biochar, an engineered PyC primarily used in agricultural applications. This alkalinity derives from alkaline surface functional groups on the aromatic PyC structure, as well as carbonates, and other inorganic moieties in the ash fraction.^45,46^ Metals, which can occur in the ash fraction, are known to interfere with the functioning of microbial cells by blocking the active center of enzymes.^19^ However, our results indicate that PyC only slightly affected the concentration of three of 24 metals quantified (see Figure S1 and Tables S1 and S2). This may be because the PyC used was one year old prior to its deployment in our experiment and, therefore, was depleted from the easily dissolvable ash fraction during field aging. A slight increase in the aqueous concentration was observed for Mn and Rb, which were likely released from the PyC. The aqueous concentration of Zn decreased in the presence of PyC, which is consistent with the increased pH and previous reports on the immobilization of Zn by engineered PyC such as biochar.^47^

**Figure 2:**
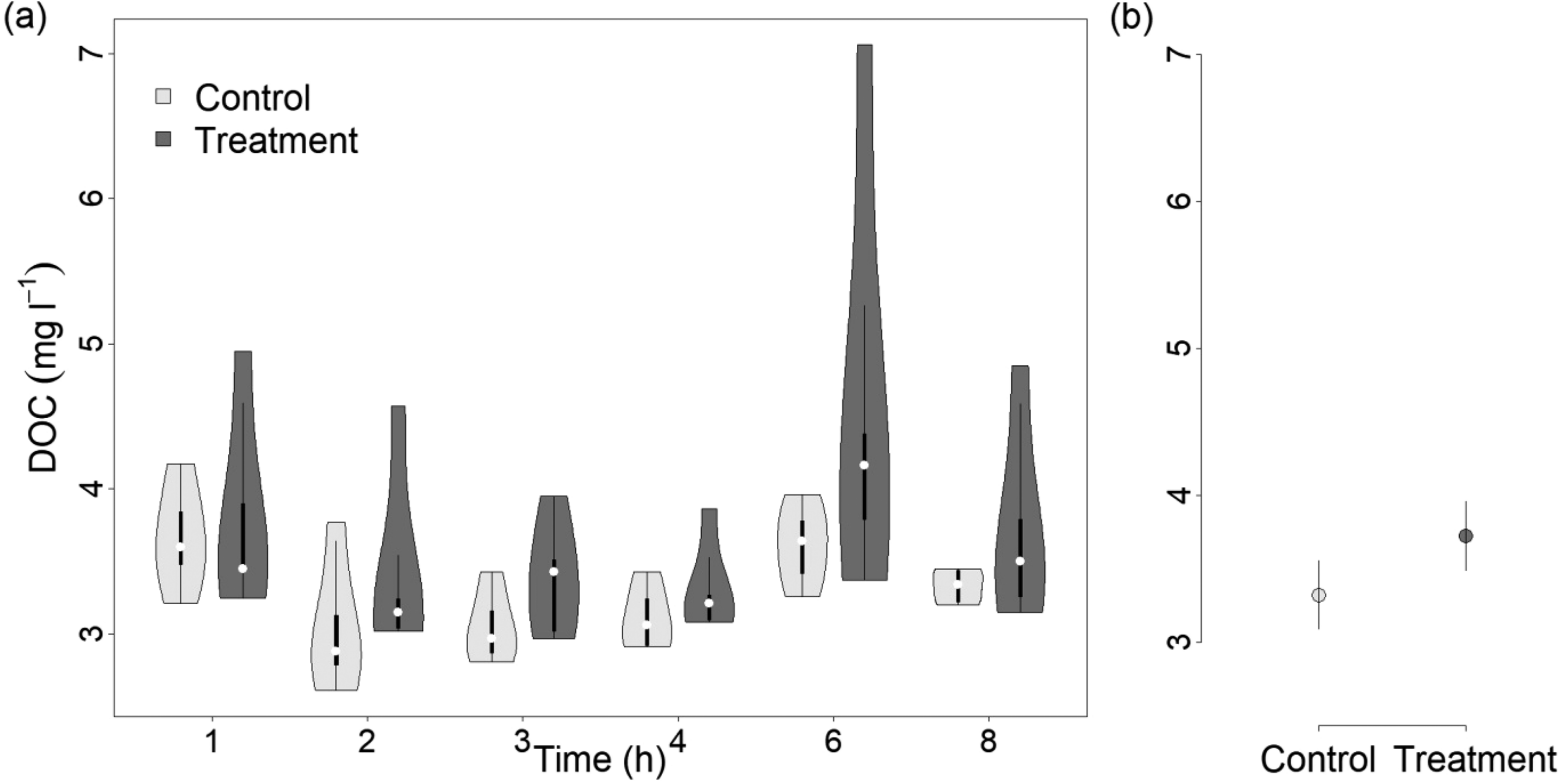
(a) DOC concentrations over time in the control and treatment flumes. White dots in box plots represent median values. (b) Mean predicted DOC concentrations based on Gaussian process models for control (light gray) and treatment (dark gray) flumes with a 90% confidence interval (CI). Effect size of 0.4 with a 90% CI (0.07, 0.73).

DOC concentrations across all time points ranged from 2.61 to 4.17 mg L^–1^ and from 2.97 to 7.06 mg L^–1^ in the control and treatment flumes, respectively. Overall, the DOC concentration slightly increased with PyC addition (Figure 2a,b). DOC concentrations were higher in the treatment (mean predicted DOC of 3.72 ± 0.14 mg L^–1^) than in the control (mean predicted DOC of 3.32 ± 0.14 mg L^–1^) flumes, with an overall effect size of 0.40 ± 0.20 mg L^–1^ based on Gaussian process regression (Figure 2b). The increases in DOC concentration in treatment flumes can be explained by leaching of pyrogenic organic matter from wildfire charcoals, as also observed in previous laboratory studies.^18,48,49^

Our results indicate that leaching of DOC from PyC and its physical disintegration exceed the adsorption of riverine DOC to PyC under conditions such as those studied here. Additionally, our measurements are probably a conservative estimate of leaching of DOM from PyC in the river water, due to the lower turbulence in flumes compared to the river.

The amount of DOC released from filter bags in the field experiments was estimated as
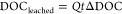
3where *Q* is the flume discharge, *t* the duration of the experiment, and ΔDOC the modeled treatment effect. These calculations indicate that over the 8 h duration of the experiment, at least 89.45 ± 0.30 mg of DOC was leached in each flume. As this calculation assumes no stream DOC was adsorbed, this is a very conservative estimation that corresponds to 0.40 ± 0.01 mg of C/g of PyC.

Under laboratory conditions, each gram of PyC in the filter bag released 0.84 ± 0.01 mg of total organic carbon (TOC) over 8 h (see Table S3), which would correspond to 189.60 ± 1.80 mg of TOC in each treatment flume. Considering this, particulate organic carbon (POC) was computed as TOC_lab_ – DOC_flume_ and amounted to 0.44 ± 0.01 mg of C/g of PyC. This represents a conservative estimate of POC as water turbulences and dilution in the flumes are expected to be greater than during the laboratory experiment, which could increase the level of POC release. On the basis of these results, POC also increased upon addition of wildfire char. This suggests that the original pieces of charcoal underwent partial disintegration, leading to small particles from the wildfire charcoal being mobilized into river water, which was confirmed by the visual deposition of small PyC particles on the biofilm at the end of the field experiment (see Figure S3).

Mean POC (TOC_lab_ – DOC_flume_) and DOC inputs were similar in size, amounting to approximately 0.44 ± 0.01 and 0.40 ± 0.01 mg of C/g of PyC, respectively. Previous studies estimated global PyC fluxes and found that the POC/DOC ratio is approximately 1.5,^2,9,50^ which is in the range of our findings. Our results show that a considerable amount of carbon can be released from aged PyC particles in dissolved and particulate forms.

### 3.2 Addition of Pyrogenic Carbon Changes the DOM Composition

**Figure 3:**
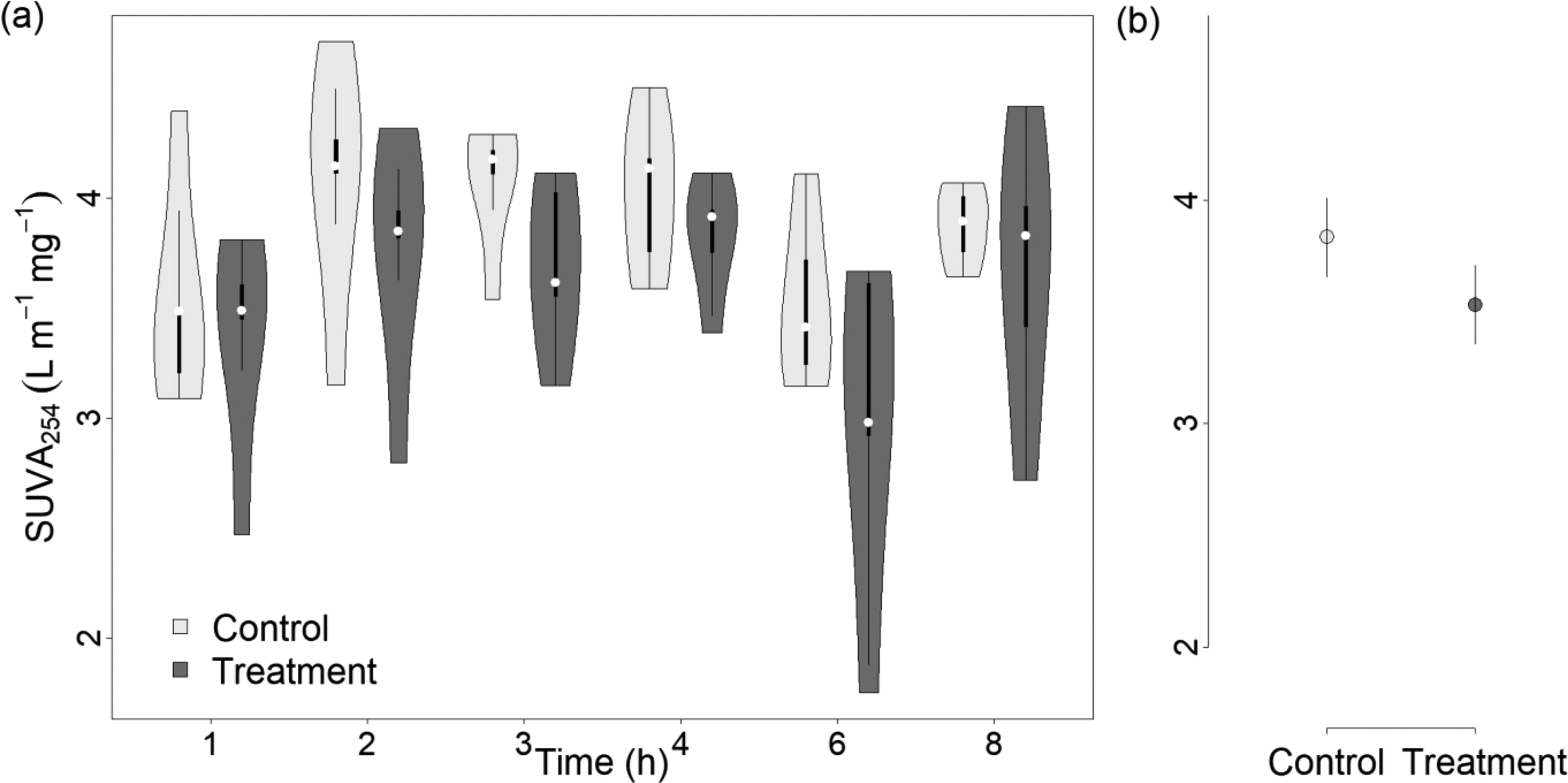
(a) SUVA_254_ over time in the control and treatment flumes. (b) Mean predicted SUVA_254_ based on Gaussian process models for control (light gray) and treatment (dark gray) flumes with a 90% CI. Effect size of −0.31 with a 90% CI (−0.56, −0.05).

SUVA_254_, which correlates strongly with aromaticity,^29^ ranged from 3.09 to 4.71 in the control and from 1.75 to 4.41 in the treatment flumes. Hence, SUVA_254_ was consistently decreased in the presence of PyC, as confirmed by Gaussian process regression, which found lower SUVA_254_ in the treatment flumes (mean predicted SUVA_254_ of 3.53 ± 0.11) than in the control flumes (mean predicted SUVA_254_ of 3.84 ± 0.11) with an overall effect size of −0.31 ± 0.15 (Figure 3).

PyC consists of both labile and highly aromatic recalcitrant fractions, with the labile fraction being less aromatic and more easily mobilized and degraded by microbes.^49^ In contrast, the highly aromatic fraction is expected to remain longer in the particulate form, constituting a strong sorbent for other aromatic DOM compounds. The notion that labile compounds with low aromaticity are leached from PyC is supported by measured low SUVA_254_ values (2.47 ± 0.17) in Milli-Q water leaching experiments (see section 2.3).

The reduced SUVA_254_ values in treatment flumes (Figure 3) could additionally suggest that selective sorption of riverine DOM by PyC simultaneously removed aromatic compounds from the water. This would be in line with recent studies showing that sorption of DOM to PyC particles increases with DOM aromaticity and can be hindered by steric effects, excluding very large DOM molecules from reaching certain sorption sites within the charcoal particles.^26,51^

**Figure 4:**
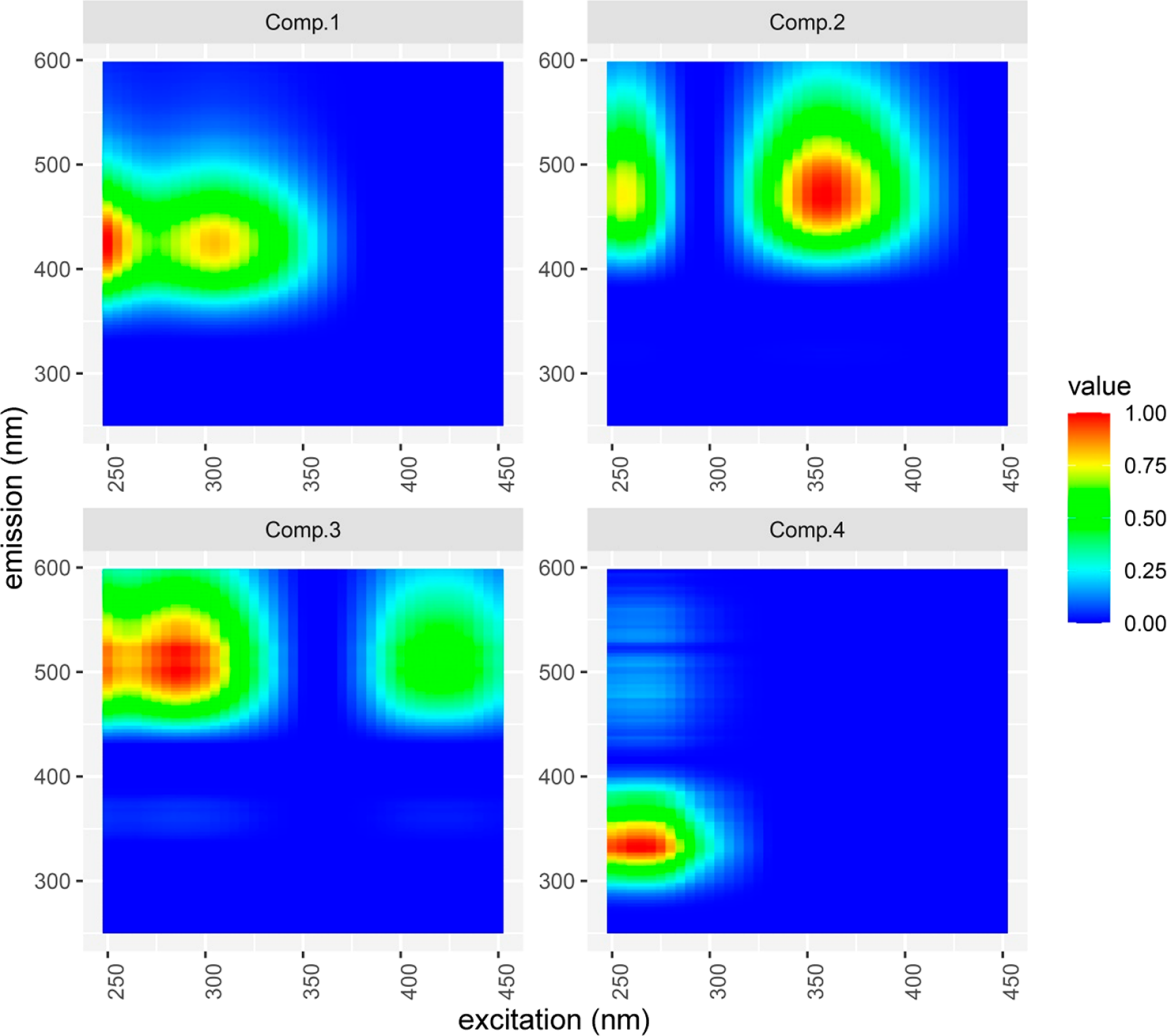
Excitation emission matrices (EEMs) for all four PARAFAC components, modeled from all samples. C1 has its excitation maximum at 250 nm and its emission maximum at 425 nm. C2 has its excitation peak at 360 nm and its emission peak at 465 nm. C3 and C4 have their excitation peaks at 285 and 265 nm and their emission peaks at 500 and 335 nm, respectively.

PARAFAC, a modeling approach used to unravel chemical signatures from EEMs, resulted in the detection of four components (see Figure 4). The PARAFAC report can be found online under the name “LimnicFires” at OpenFluor (https://openfluor.lablicate.com), which is a platform for published PARAFAC models.^52^ No significant differences were observed due to additions of PyC in any of the four components (see Figure S4).

C1–C3 are humic-like components, whereas C4 is a protein-like component.^30,53−57^ C2 is possibly microbial humic-like on the basis of the results of Yamashita and colleagues.^58^ Although not clearly detectable, PARAFAC components suggest that there was a slight increase in mostly humic-like chemical compounds following PyC addition (see Figure S4). This notion is supported by the measured total fluorescence that, albeit not normalized for DOC, slightly increased due to PyC addition (see Figure S5a). When normalized to DOC, patterns invert, indicating that a large part of the effect of PyC on total fluorescence derives from the increased DOC concentration (see Figure S5b). However, further investigations of PARAFAC components from similar experiments at larger scales and possibly at varying ranges of PyC concentrations are needed to confirm these interpretations.

### 3.3 Addition of Pyrogenic Carbon Affects Enzymatic Activities

**Figure 5:**
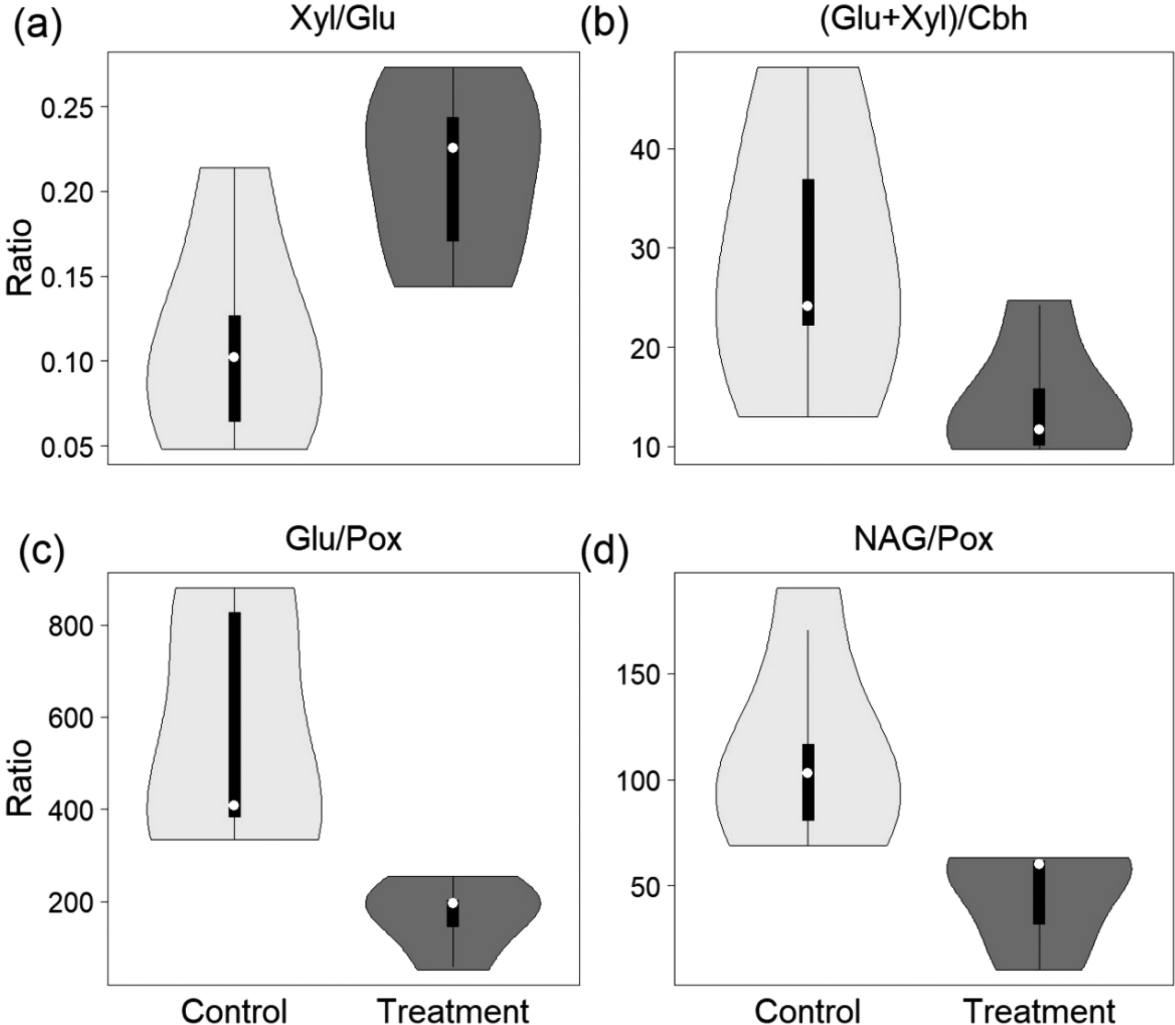
Enzyme ratios without (light gray) and with (dark gray) PyC addition. Abbreviations: Xyl, β-xylosidase; Glu, β-glucosidase; Cbh, cellobiohydrolase; Pox, phenol oxidase; NAG, β-*N*-acetylglucosaminidase. (a) Xyl/Glu ratio. (b) (Glu + Xyl)/Cbh ratio. (c) Glu/Pox ratio, i.e., recalcitrant index. (d) NAG/Pox ratio.

Oxidation and hydrolysis are two key processes for DOM degradation. Hydrolytic enzymes degrade non-aromatic DOM structures, while oxidative enzymes additionally degrade aromatic DOM structures.^59^ PyC addition significantly decreased the ratio of hydrolytic to oxidative enzymes, indicating a shift toward degradation of aromatic over non-aromatic compounds (see Figure 5 and Figure S6).

The ERs Xyl/Glu, (Glu + Xyl)/Cbh, Glu/Pox, and NAG/Pox were affected by PyC addition (Figure 5). Xly/Glu increased upon PyC addition (*t* test, df = 4, *p* = 0.03), indicating a preferential use of large polymeric carbon compounds in comparison to the control flumes. The (Glu + Xyl)/Cbh ratio decreased with PyC addition (*t* test, df = 4, *p* = 0.08), indicating the reduced use of readily available polysaccharides in comparison to complex polysaccharides. Glu/Pox, also termed the recalcitrance index, decreased with PyC addition (*t* test, df = 4, *p* = 0.03), pointing to the increased use of polyphenolic lignin-like compounds in comparison to readily available compounds such as cellobiose or small oligomers.^37^ In addition, the increased use of a rather recalcitrant material is supported by an increased Pox activity and a decreased Glu activity due to PyC addition (see Table S4), although these activities need to be interpreted carefully as they are not normalized for biomass. Lastly, NAG/Pox decreased with PyC addition (*t* test, df = 4, *p* = 0.03), indicating an increased use of polyphenolic lignin-like compounds over chitin-derived compounds. Furthermore, the ER variability decreased following PyC addition, especially for the ERs Glu/Pox and NAG/Pox (Figure 5c,d).

Importantly, the large number of particles that settled on the biofilm as observed microscopically (see Figure S3) might additionally increase the effects of PyC on enzymatic activity via direct interaction with the biofilm matrix, including the exposure of microbiota to the highly aromatic chemical structure of PyC particles that might have affected the organic matter pool directly available in the biofilm. Particulate-bound aromatic organic matter may have induced an increased activity of Pox (Figure 5c,d) compared to that of purely hydrolytic ERs (Figure 5a,b), even though such aromatic compounds are generally highly recalcitrant as was recently confirmed in a laboratory incubation experiment with PyC-derived DOM.^18^ Furthermore, PyC could possibly cause oxidative stress to the biofilms via persistent free radicals that have recently been measured in wildfire-derived PyC.^28^

Observed alterations of ERs in benthic biofilms with PyC addition can be explained by the combined effect of changes in DOC quantity, DOM quality, and pH. These findings are in line with studies of potential drivers of ER variabililty.^24,35,38,60^ For instance, in a meta-analysis comparing terrestrial, marine, and freshwater ecosystems, Arnosti and colleagues^60^ found that pH is more important in controlling enzymatic activities in freshwater ecosystems than in marine environments. Freixa and colleagues^24^ related enzymatic activities in a longitudinal river continuum to the change in DOM composition from up- to downstream and found that enzymatic activities reflect a transition from allochthonous to autochthonous DOM along the river continuum.

In this study, which used field-aged wildfire charcoal that was likely depleted of mobile metals due to leaching in the field, the small changes in metal concentration observed very likely did not have an effect on enzyme activities. For example, Zn has been shown to inhibit the β-glucosidase activity of in-stream biofilms.^61^ Thus, on the basis of the slightly lower Zn concentration measured in the treatment flumes, PyC could be expected to lead to an increase in the recalcitrant index (i.e., Glu/Pox ratio). However, we observed the opposite pattern (Figure 5c), likely because other factors, including the DOM composition, were more important for biofilm functioning. Future experiments using ash-rich and fresh PyC will be necessary to elucidate effects on biofilm functioning of metals that can be mobilized during wildfires.

The ERs in this study indicate that PyC addition led to an overall compositional change of DOM toward lower biodegradability, although DOC increased (Figure 2) and DOM aromaticity decreased (Figure 3). This is in line with studies of non-wildfire-affected streams reporting changes in enzymatic activity with the dependence of OM substrate composition.^24^ At first, it may appear contradictory that upon addition of PyC, ERs indicate a decrease in easily assimilable DOM while SUVA_254_ indicates a decreased DOM aromaticity. Aromaticity, however, is not the only DOM property linked to recalcitrance and decreased degradability. For instance, molecular size, aqueous solubility, oxidation state, molecular complexity, and the lack of N-containing substituents are also linked to the recalcitrance of organic matter.^62^

Here we show that in this field-based experiment, the input of PyC increases riverine DOC concentrations, changes DOM composition, and modifies biofilm enzyme activities. Our results therefore indicate that PyC can alter fluvial carbon cycling, albeit the magnitude and transferability of our results need to be investigated further, especially in other rivers and at larger spatial and temporal scales.

## 4 Implications

Water erosion and colloidal transport of PyC into limnic systems can cause substantial changes in DOC concentration and DOM composition. We here report on the release of organic matter from PyC with the potential simultaneous selective adsorption of native aromatic substances from riverine DOM in a natural river system. Our results indicate that PyC, in both particulate and dissolved form, affects enzymatic activities in benthic biofilms, especially for oxidative enzymes. PyC addition increased pH, which can also play a role in altering enzymatic activities. Lastly, deposition of particulate PyC directly on the biofilm surface brings the PyC into close contact with the biofilm matrix, potentially modulating the aforementioned effects. Overall, our results suggest that inputs of PyC into freshwater can directly affect enzymatic activities, thus altering in-stream benthic biofilm functioning and carbon cycling. Our in-stream flume approach, applied here for the first time, can be adapted to study a diverse range of rivers, enabling a more comprehensive understanding of the effects of wildfires on riverine carbon cycling. Further experiments in which the amount of PyC is varied could be used to construct dose–response curves, and changing the exposure time could help gain insights into changes in enzymatic activity over time. In addition, in future experiments, ^13^C-labeled PyC could be used to differentiate DOM from PyC from riverine DOM, potentially providing valuable additional insights into the mechanism at play.

## Supporting Information Available

Measured metal concentrations, measured environmental parameters in the field, microscopy, parallel factor analysis, and experimental details of extracellular enzymatic activities (PDF)
