# Supplementary material for: Wildfire-Derived Pyrogenic Carbon Modulates Riverine Organic Matter and Biofilm Enzyme Activities in an In Situ Flume Experiment
Source: ACS ES T Water. 2021 Jun 25;1(7):1648–56. doi: 10.1021/acsestwater.1c00185 (PMC8276270; doi:10.1021/acsestwater.1c00185)
Supplement: Supplementary file 1 — ew1c00185_si_001.pdf [file ew1c00185_si_001.pdf]

Wildfire-derived pyrogenic carbon modulates riverine organic matter and biofilm enzyme activities in an in-situ flume experiment

Supporting Information

Lukas Thuile Bistarelli,[1] Caroline Poyntner,[2] Cristina Santín,[3,4] Stefan Helmut Doerr,[5] Matthew V. Talluto,[1] Gabriel A. Singer,[1] Gabriel Sigmund[6]*

[1] Institute of Ecology, University of Innsbruck, Technikerstraße 25, 6020 Innsbruck, Austria

[2] Institute of Microbiology, University of Innsbruck, Technikerstraße 25, 6020 Innsbruck, Austria

[3] Research Unit of Biodiversity, Spanish National Research Council (CSIC), E-33600 Mieres, Spain

[4] Swansea University, Department of Biosciences, Singleton Park, Swansea SA2 8PP, UK

[5] Swansea University, Department of Geography, Singleton Park, Swansea SA2 8PP, UK

[6] University of Vienna, Department of Environmental Geosciences, Centre for Microbiology and Environmental Systems Science, Althanstraße 14, 1090 Wien, Austria

Corresponding author: Gabriel Sigmund, University of Vienna, Environmental Geosciences, Centre for Microbiology and Environmental Systems Science, Althanstraße 14, 1090 Wien, AT.
e-mail: gabriel.sigmund@univie.ac.at

1. Metals

Sampling for metals took place 1, 4, and 8 h after the beginning of the experiment. Water samples were filtered through pre-washed 0.2 µm Minisart syringe filters (Sartorius, Germany) into sterile 15 ml Falcon tubes and acidified with HNO_3 thereafter. Concentrations of 24 metals (see Table S1 for the whole description) were quantified using an inductively coupled plasma optical emission spectrometer (ICP-OES, Agilent 5110, United States) and a quadrupole inductively coupled plasma mass spectrometer (ICP-MS, Agilent 7900, United States).

Out of the 24 metals analyzed, we found that concentrations of three changed between treatment and control flumes. For Manganese, Zinc and Rubidium the effect was strongest at the beginning of the experiment and decreased over time (Fig. S1). Manganese and Rubidium concentrations increased with PyC addition, whereas Zinc concentrations decreased following PyC addition. All differences were evaluated using the Gaussian Process regression described in the main manuscript. The overall effect sizes and the 90% credible intervals (CI) can be found in table S2. However, the differences in concentration are not expected to produce observable biological effects and were thus not further explored (see main manuscript).

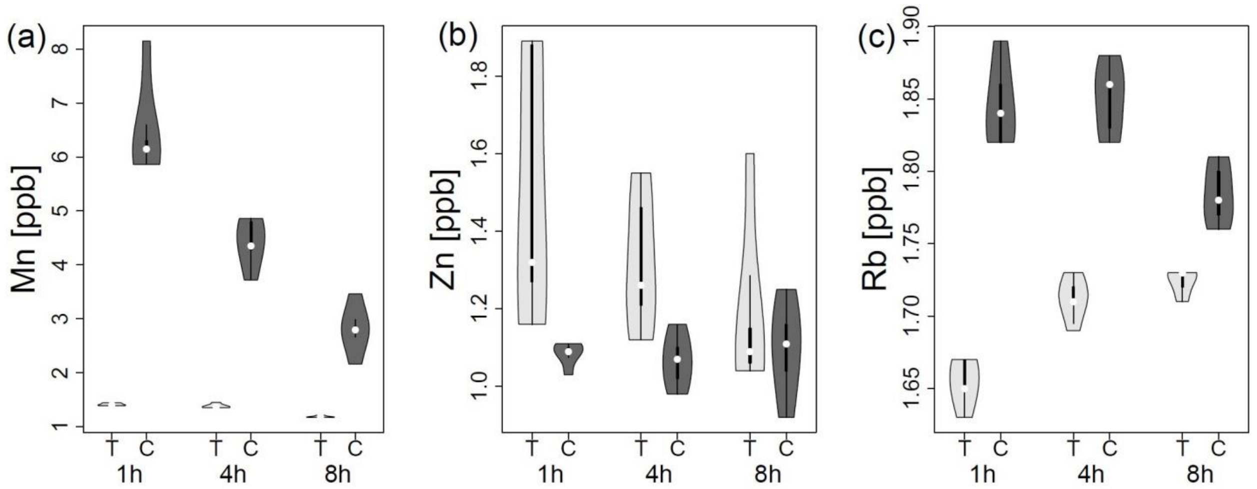

Figure S1: Violin plots of treatment (dark grey) and control (light grey) values at each sampling time (1, 4, and 8 h after the beginning of the experiment) for Manganese (a), Zinc (b), and Rubidium (Rb)

Table S1: Mean metal concentrations and standard error (SE) derived from five replicate measurements (one for each flume) for treatment and control during each of the three samplings: 1, 4 and 8 h.

Time (h)	**1**				**4**				**8**			
Treatment	**Treatment**		**Control**		**Treatment**		**Control**		**Treatment**		**Control**	
Measure	**mean**	**SE**	**mean**	**SE**	**mean**	**SE**	**mean**	**SE**	**mean**	**SE**	**mean**	**SE**
Li [ppb]	1.78	0.01	1.75	0.02	1.80	0.02	1.82	0.03	1.78	0.02	1.81	0.01
Na [ppb]	6002.80	21.53	5968.20	13.00	5975.80	53.10	5929.40	19.54	5683.60	33.06	5672.80	42.26
Mg [ppb]	3126.80	13.89	3116.20	4.73	2995.20	29.97	2980.40	14.83	2814.20	18.16	2799.40	22.54
Al [ppb]	37.36	0.24	36.46	0.38	39.96	0.34	40.06	0.16	41.82	0.53	41.00	0.33
Ca [ppb]	11751.20	56.61	11705.40	20.36	11479.00	112.14	11447.00	49.48	10968.00	66.62	10920.00	89.24
Sc [ppb]	1.10	0.01	1.19	0.03	1.09	0.00	1.07	0.01	1.04	0.01	1.03	0.00
Mn [ppb]	6.51	0.42	1.41	0.01	4.40	0.21	1.38	0.02	2.81	0.21	1.18	0.01
Fe [ppb]	53.02	0.55	51.34	0.80	56.14	0.59	56.86	0.39	58.50	0.48	56.34	0.46
Co [ppt]	48.10	0.86	49.28	0.89	46.92	0.72	45.64	0.98	43.52	1.20	41.40	0.63
Zn [ppb]	1.08	0.01	1.50	0.16	1.07	0.03	1.32	0.08	1.10	0.06	1.19	0.10
Rb [ppb]	1.85	0.01	1.65	0.01	1.85	0.01	1.71	0.01	1.78	0.01	1.72	0.00
Sr [ppb]	48.42	0.17	48.94	0.16	47.42	0.17	47.34	0.12	45.12	0.15	45.38	0.12
Y [ppb]	0.12	0.00	0.12	0.00	0.12	0.00	0.12	0.00	0.12	0.00	0.12	0.00
Mo [ppb]	0.17	0.00	0.17	0.00	0.16	0.00	0.16	0.00	0.16	0.00	0.16	0.00
Ba [ppb]	10.13	0.06	10.08	0.04	9.58	0.03	9.55	0.03	9.12	0.02	9.17	0.04
La [ppt]	67.54	0.65	68.22	0.90	71.96	0.84	71.62	0.39	73.46	0.75	73.66	0.72
Ce [ppt]	105.40	1.25	103.36	2.54	110.80	1.66	112.20	0.97	113.00	0.55	113.40	0.98
Pr [ppt]	19.04	0.30	18.88	0.54	20.64	0.32	19.78	0.19	20.86	0.15	20.48	0.24
Nd [ppt]	84.02	1.90	80.68	0.65	87.22	0.68	88.98	1.34	89.54	1.08	87.68	1.34
Sm [ppt]	21.82	0.61	20.96	0.93	22.38	0.46	21.84	0.78	22.08	1.21	21.88	0.72
Gd [ppt]	22.02	0.42	21.48	0.25	24.76	0.32	23.80	0.34	23.78	0.57	23.52	0.33
Dy [ppt]	20.14	0.63	20.38	0.32	21.82	0.60	21.78	0.19	21.92	0.66	22.32	0.22
Pb [ppt]	66.20	0.29	325.58	189.74	68.56	1.14	70.00	1.03	68.86	0.63	783.60	164.92
U [ppt]	48.54	0.62	47.36	0.45	47.76	0.33	48.64	0.45	44.52	0.22	44.96	0.43

Table S2: Overall effect sizes of the PyC addition. Median and 90% Credible Intervals are given for each metal in the control and treatment flumes.

		**Median**	**90% CI**
**Mn**	Control	1.33	(0.78, 1.90)
	Treatment	4.55	(4.00, 5.01)
**Zn**	Control	1.34	(1.24, 1.43)
	Treatment	1.08	(0.99, 1.18)
**Rb**	Control	1.70	(1.68, 1.71)
	Treatment	1.83	(1.81, 1.84)

2. Environmental Parameters of In-Stream Flume Water

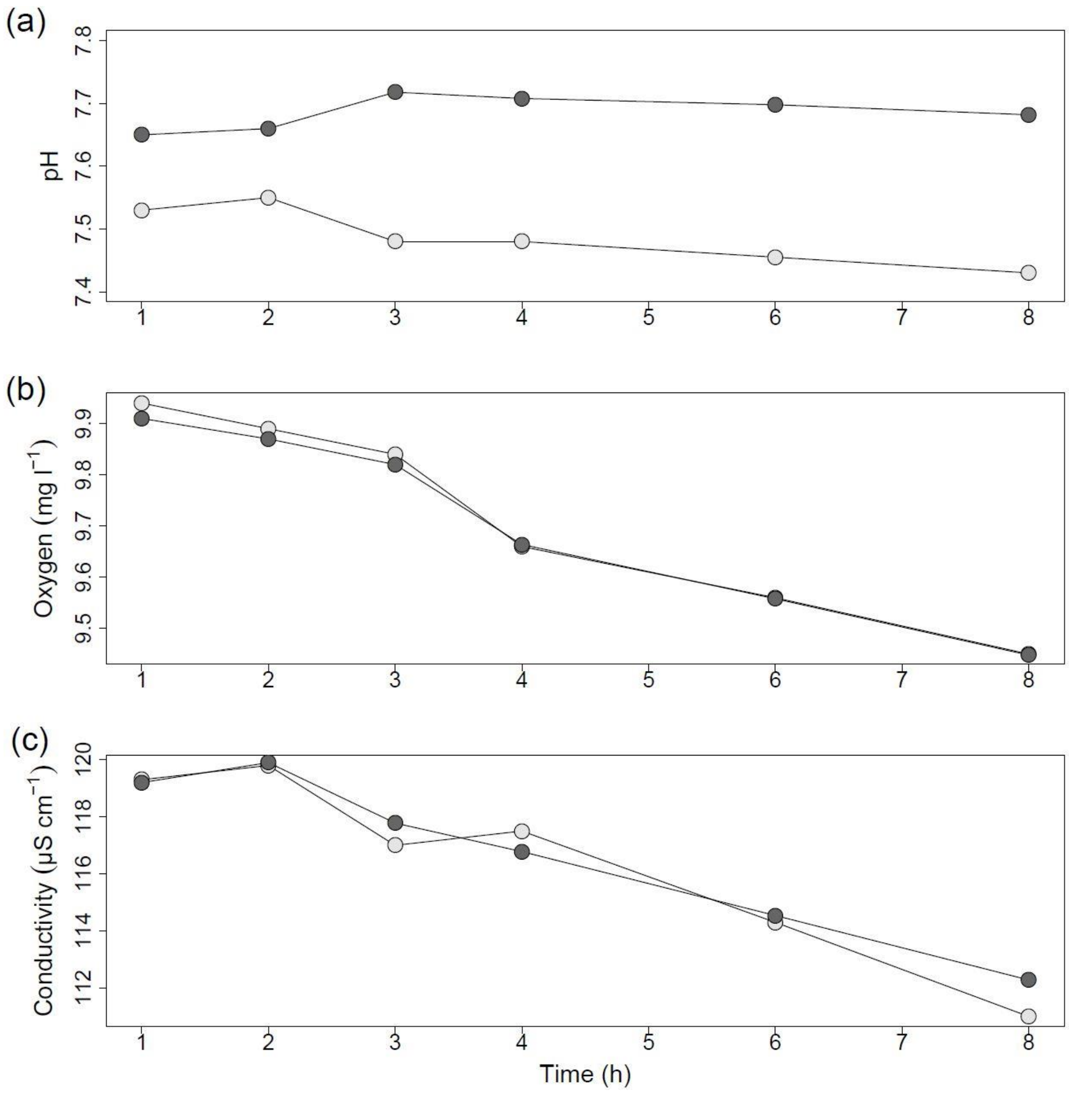

Figure S2: Physico-chemical parameters over the duration of the experiment. (a) pH, (b) oxygen, and (c) conductivity. Light grey dots represent the control, dark grey dots the treatment.

Table S3: Mean and standard error (SE) of total organic carbon in mg l^{-1} derived from 8h laboratory experiments in 1 l Schott bottles containing river water (control) or 1 filter bag with 15 g PyC (treatment)

	**mean**	**SE**
**Control**	8.50	0.04
**Treatment**	21.14	0.05

3. Microscopy

Next to the tiles, microscope slides were laid out in the stream bed from the 14th of June 2019 until the 12th of July 2019 resulting in colonization of local biofilm communities on the slide surface. The slides were placed at the bottom of the flumes during the treatment and transported in the flume water to the lab for light microscopy (Olympus BX51, Japan). Particulate PyC sedimentation on biofilm surfaces was observed (Fig. S5 a and b). The microbial cells of the biofilm did not show any notable differences between treatment and control (Fig. S5 b and c).

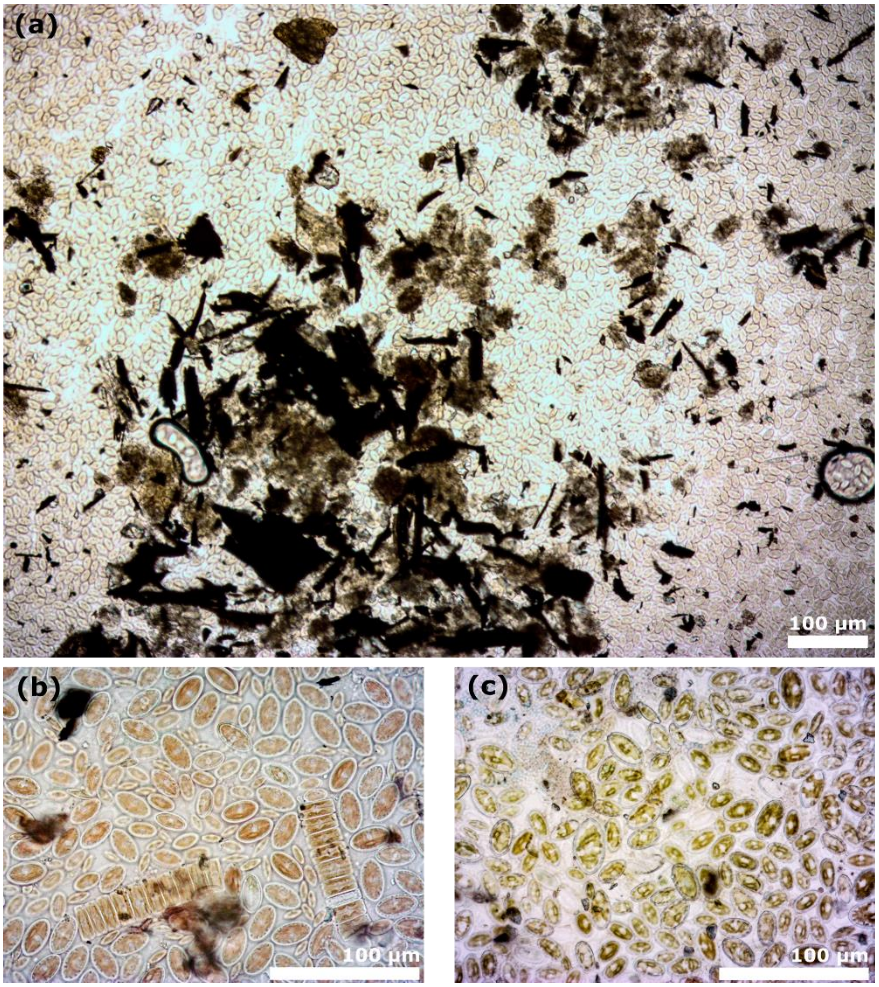

Figure S3: Light microscope pictures taken at the end of the 8 h experiment. Treatment samples are shown in (a) and (b), a control sample is shown in (c). The dark structures in a) and b) are sedimented PyC particles. The difference in cell coloring is the result of different exposure conditions for improving the imaging of samples containing PyC.

4. Parallel Factor Analysis

Overall effect sizes in the Gaussian process regressions where too small to deduce any treatment effect. Nevertheless, C1 and C3 showed a trend towards a higher signal in flumes with PyC addition.

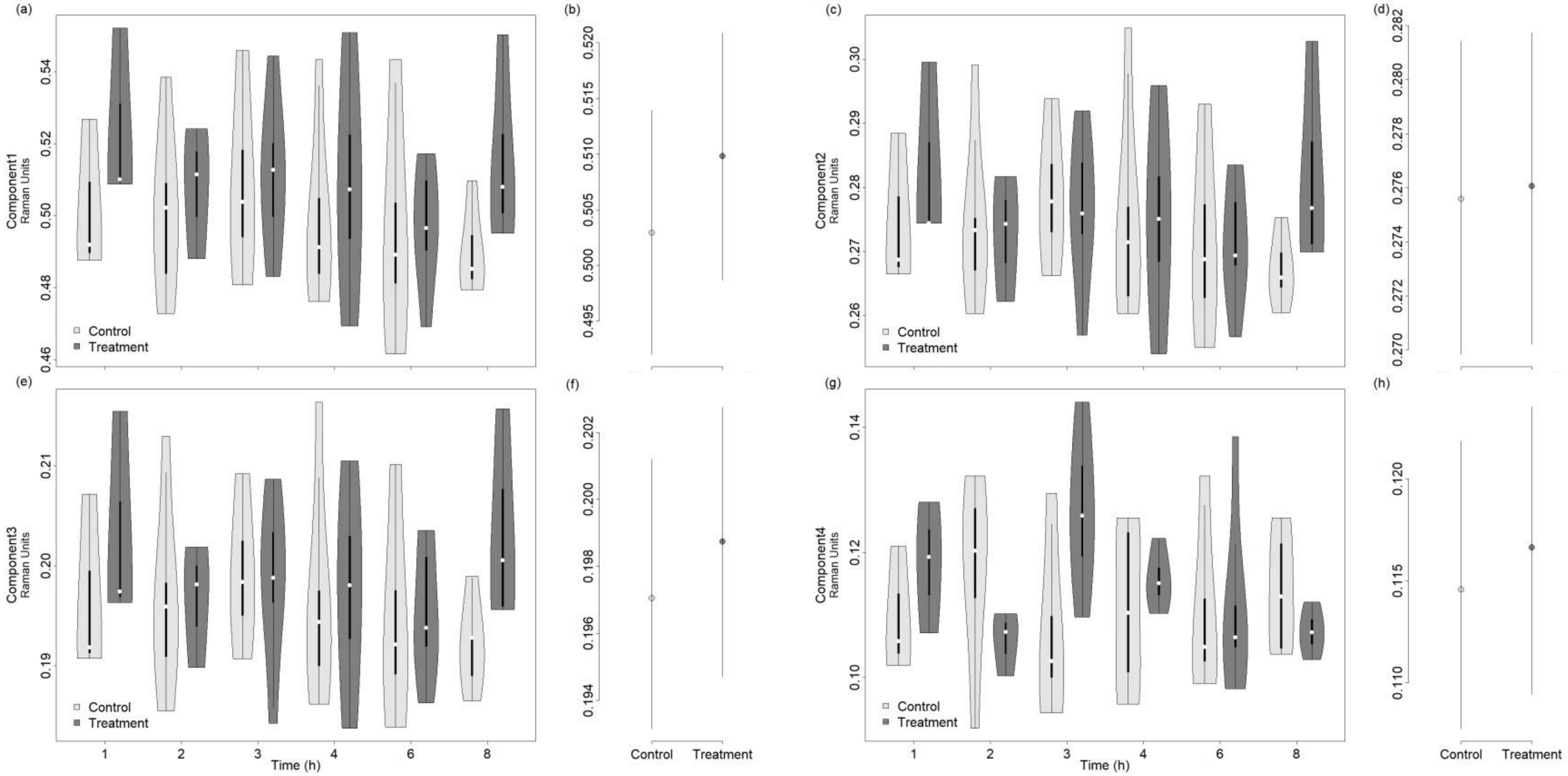

Figure S4: a) C1 over time in the control and treatment flumes. b) Mean predicted C1 based on Gaussian process models for control (light grey) and treatment (dark grey) flumes with 90 % CI. c) C2 over time in the control and treatment flumes. d) Mean predicted C2 based on Gaussian process models for control (light grey) and treatment (dark grey) flumes with 90 % CI. e) C3 over time in the control and treatment flumes. f) Mean predicted C3 based on Gaussian process models for control (light grey) and treatment (dark grey) flumes with 90 % CI. g) C4 over time in the control and treatment flumes. h) Mean predicted C4 based on Gaussian process models for control (light grey) and treatment (dark grey) flumes with 90 % CI.

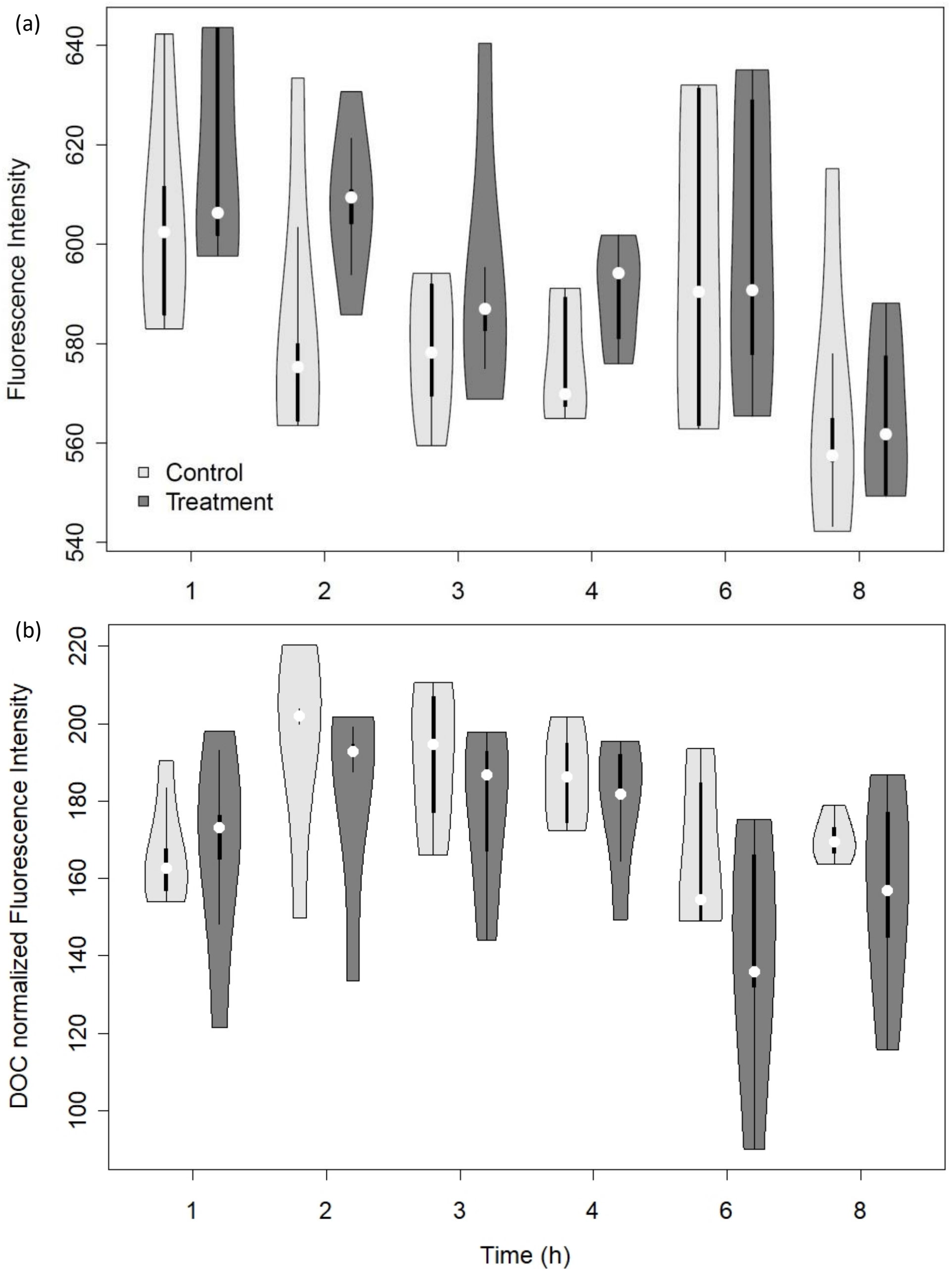

Figure S5: a) Total fluorescence intensity over the duration of the experiment. b) DOC normalized total fluorescence intensity over the duration of the experiment. Light grey represents the control flumes and dark grey the treatment flumes.

5. Extracellular Enzymatic Activities

The enzymes β-glucosidase (Glu), β-xylosidase (Xyl), cellobiohydrolase (Cbh), β-N-acetylglucosaminidase (NAG), phosphatase (Pho), and lipase (Lip) were measured using the MUF (methylumbelliferone) based artificial fluorescence substrates MUF-β-d-glucoside (Sigma-Aldrich, United States), MUF-β-d-xyloside (Sigma-Aldrich, United States), MUF-cellobioside (Sigma-Aldrich, United States), MUF-N-acetyl-β-d-glucosaminide (Sigma-Aldrich, United States), MUF-phosphate (Sigma-Aldrich, United States) and MUF-palmitate (AGscientific, United States) respectively. Leucine-aminopeptidase (Pep) was measured based on the AMC (7-amino-4-methylcoumarin) linked fluorescent substrate l-leucine-4-methyl-7-coumarinylamide (Sigma-Aldrich, United States). Phenol oxidase (Pox), as the only absorbance based artificial substrate was measured using L-DOPA (L-3,4- dihydroxyphenylalanine). [1–9]

Table S4: Mean enzyme activities and standard error (SE) derived from five replicate measurements (one for each flume) for treatment and control. Values where corrected for tile surface and dilution

	Treatment		Control	
	**mean**	**SE**	**mean**	**SE**
Peptidase	0.079663	0.010546	0.060071	0.034422
Glucosidase	0.012903	0.006900	0.031226	0.002612
Xylosidase	0.002896	0.000339	0.002839	0.000746
Cellobiohydrolase	0.001358	0.000150	0.001238	0.000434
Phosphatase	0.046747	0.006044	0.039138	0.012026
N-acetylglucosaminidase	0.003400	0.001053	0.006053	0.000740
Phenol Oxidase	0.000081	0.000003	0.000055	0.000010

Enzyme assays were prepared a-priori in a Riplate® medio deep well plate (Ritter Medical, Germany), covered with polymerase chain reaction (PCR) foil and transported frozen to the study site: Each row consisted of one enzyme replicated five times (columns 1-5), three controls (columns 6-8), one biofilm quenching well (column 9), and one abiotic degradation well (column 12). Columns 10 and 11 were used as standards where a dilution series of MUF and AMC with a final concentration of 0, 0.25, 0.5, 2.5, 5, 10, 50, and 100 µM was used. For columns 1-5, 9, and 12 100 µl of artificial substrate were used so that we achieved a final concentration of 300 µM for all fluorescence base artificial substrates and a final concentration of 1.5 mM for Pox.

Deep well plates where unfrozen right before the beginning of the enzyme assay incubation. At the end of the flume experiment we scraped the biofilm from the tiles using sterile scalpels. Thereafter the biofilm slurry was placed in a 50ml Falcon tube, diluted by adding 17.5 ml of 0.2 µm filtered stream water, and homogenized using a frother. The biofilm slurry was placed into a Dual solution® pipetting reservoir (Sigma Aldrich, United States) and 300 µl of slurry where pipetted into columns 1-9 on the well plates. The plates were sealed with the PCR foil and placed into dark and air-tight chambers that were incubated in the river under water for 1h. At the end of the incubation, we added 100 µl of glycine buffer (pH=10.4) to all fluorescence-based artificial substrates to stop the reaction. For Pox, we added 100 µl of acetate buffer (pH=5). Subsequently, deep well plates were frozen and analyzed in the lab after two days. First, to avoid particles during pipetting, plates were centrifuged at 500 rpm for 2 minutes. Thereafter, 200 µl of supernatant were transferred into black microtiter plates with transparent bottom (Greiner Bio-One, Germany). Samples were analyzed using a Spark plate-reader (Tecan Trading AG, Switzerland). MUF and AMC based artificial substrates were measured using 365/455 nm excitation/emission and 364/445 nm excitation/emission respectively. Pox was measured via absorbance readings at 460 nm. To convert the absorbance data into amount of 2, 3-dihydroindole- 5,6-quinone-2-carboxilate (DIQC; compound created after cleavage of L-DOPA) released we applied the extinction coefficient (k) following this formula: (abs sample – abs control)/k, where abs is the measured absorbance and the constant k=1.66 mM^{-1} [10].

All fluorescence-based values were corrected for tile surface (25 cm^2), dilution (17.5 ml), biofilm quenching (i.e. fluorescence alterations due to substances present in the biofilm other than the target fluorophores), and abiotic degradation (via subtracting measurements from abiotic control wells containing MilliQ water and the artificial substrate). Absorbance based values were corrected for tile surface and dilution, as described above for fluorescence-based assays. As we had no means of assessing bacterial biomass, the last step in data preparation was to compute enzyme ratios (ERs), which are dimensionless numbers and therefore biomass independent. [3,9]

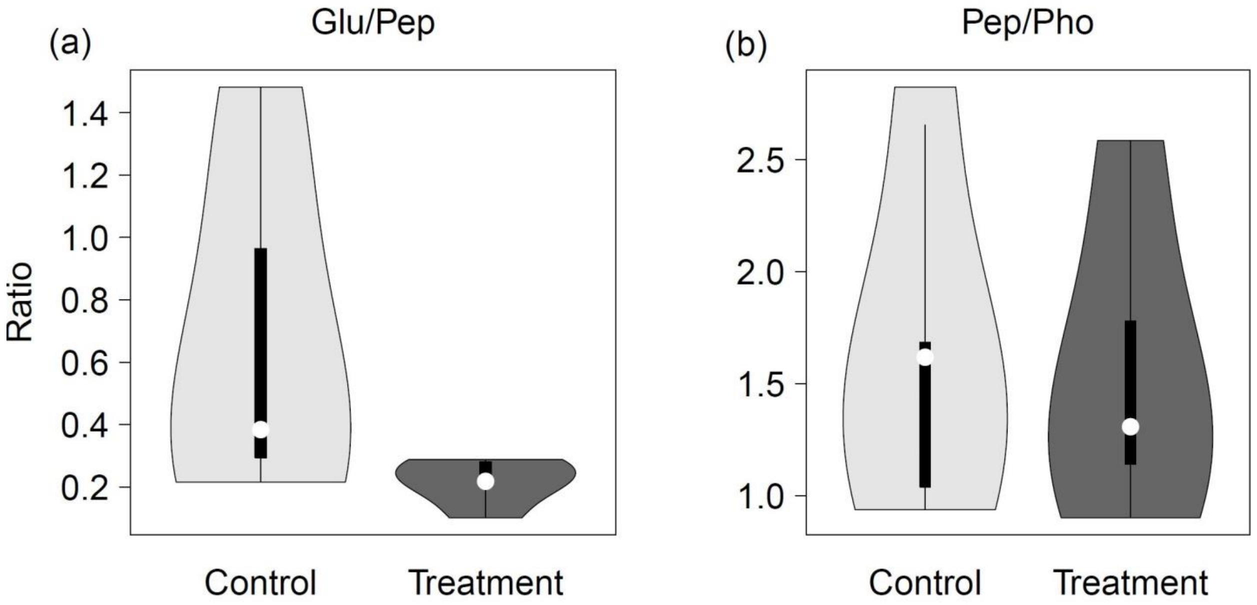

Figure S6: Violin plots for (a) Glu/Pep and (b) Pep/Pho ratios. Light grey represents values from the control flumes and dark grey values from the treatment flumes.

6. References

(1) Romani, A. M.; Sabater, S. Structure and Activity of Rock and Sand Biofilms in a Mediterranean Stream. *Ecology* **2001**, *82* (11), 3232–3245. https://doi.org/10.1890/0012-9658(2001)082[3232:SAAORA]2.0.CO;2.

(2) Romaní, A. M.; Fischer, H.; Mille-lindblom, C.; Tranvik, L. J.; Roman, A. M.; Fischer, H.; Mille-lindblom, C.; Tranvik, L. J. Interactions of Bacteria and Fungi on Decomposing Litter : Differential Extracellular Enzyme Activities. *Ecology* **2006**, *87* (10), 2559–2569. https://doi.org/10.1890/0012-9658(2006)87[2559:IOBAFO]2.0.CO;2.

(3) Ylla, I.; Peter, H.; Romaní, A. M.; Tranvik, L. J. Different Diversity-Functioning Relationship in Lake and Stream Bacterial Communities. *FEMS Microbiol. Ecol.* **2013**, *85* (1), 95–103. https://doi.org/10.1111/1574-6941.12101.

(4) Romaní, A. M.; Artigas, J.; Ylla, I. Extracellular Enzymes in Aquatic Biofilms: Microbial Interactions versus Water Quality Effects in the Use of Organic Matter. *Microb. biofilms* **2012**, 153–174.

(5) Sinsabaugh, R. L.; Follstad Shah, J. J.; Hill, B. H.; Elonen, C. M. Ecoenzymatic Stoichiometry of Stream Sediments with Comparison to Terrestrial Soils. *Biogeochemistry* **2012**, *111* (1–3), 455–467. https://doi.org/10.1007/s10533-011-9676-x.

(6) Romaní, A. M.; Vázquez, E.; Butturini, A. Microbial Availability and Size Fractionation of Dissolved Organic Carbon After Drought in an Intermittent Stream: Biogeochemical Link Across the Stream–Riparian Interface. *Microb. Ecol.* **2006**, *52* (3), 501–512. https://doi.org/10.1007/s00248-006-9112-2.

(7) Romaní, A. M.; Guasch, H.; Munoz, I.; Ruana, J.; Vilalta, E.; Schwartz, T.; Emtiazi, F.; Sabater, S. Biofilm Structure and Function and Possible Implications for Riverine DOC Dynamics. *Microb. Ecol.* **2004**, *47* (4), 316–328. https://doi.org/10.1007/s00248-003-2019-2.

(8) Freixa, A.; Ejarque, E.; Crognale, S.; Amalfitano, S.; Fazi, S.; Butturini, A.; Romaní, A. M. Sediment Microbial Communities Rely on Different Dissolved Organic Matter Sources along a Mediterranean River Continuum. *Limnol. Oceanogr.* **2016**, *61* (4), 1389–1405. https://doi.org/10.1002/lno.10308.

(9) Sinsabaugh, R. L.; Follstad Shah, J. J. Ecoenzymatic Stoichiometry of Recalcitrant Organic Matter Decomposition: The Growth Rate Hypothesis in Reverse. *Biogeochemistry* **2011**, *102* (1–3), 31–43. https://doi.org/10.1007/s10533-010-9482-x.

(10) Hendel, B.; Sinsabaugh, R. L.; Marxsen, J. Lignin-Degrading Enzymes: Phenoloxidase and Peroxidase. In *Methods to Study Litter Decomposition: A Practical Guide*; Bärlocher, F., Gessner, M. O., Graça, M. A. S., Eds.; Springer International Publishing: Cham, 2020; pp 425–431. https://doi.org/10.1007/978-3-030-30515-4_46.
